# Deploying Health Care Providers During the COVID-19 Pandemic

**DOI:** 10.1017/dmp.2021.116

**Published:** 2021-04-19

**Authors:** Aryeh Shander, Jay Mesrobian, Jeffrey Weiss, Mazyar Javidroozi

**Affiliations:** 1 Department of Anesthesiology, TeamHealth, Knoxville, TN, USA; 2 Department of Anesthesiology, Critical Care and Hyperbaric Medicine, Englewood Hospital and Medical Center, Englewood, NJ, USA

**Keywords:** COVID-19, deployment, novel coronavirus, surge capacity

## Abstract

As the COVID-19 pandemic runs its course around the globe, a mismatch of resources and needs arises: In some areas, health care systems are faced with an increased number of COVID-19 patients potentially exceeding their capacity, whereas, in other areas, health care systems are faced with procedural cancelations and a drop in demands. TeamHealth in Knoxville, Tennessee, a multidisciplinary health care organization, was able to roll out a systemic approach to redeploy its clinicians practicing in the fields of emergency medicine, hospital medicine, and anesthesiology from areas of less need (faced with reduced or no work) to areas outside of their normal practice facing immediate need.

## Introduction

As the COVID-19 pandemic runs its course around the globe, health care systems have braced for incoming waves of patients at various times. Soon after the early surge in Wuhan, China, Northern Italy became the epicenter of the pandemic, straining the local health care capacity.^1^ Other regions in the Europe and the United States were to follow. In the United States, there has been great variation in the dynamics of the pandemic and caseloads and peak resource use across the states.^2^ Resource use and demand at more local levels – counties and individual hospitals – can follow dynamics of their own.

Many regions have seen subsequent resurgence of COVID-19 infections following the initial waves. As a consequence, at any given time, various regions could be faced with varying degrees of caseload, some potentially exceeding the local health care capacity. The surge in hospital admissions and need for critical care beds can be addressed by increasing the surge capacity of intensive care units (ICUs) through various strategies, including internal restructuring of bed assignments, as well as adjusting staffing numbers and ratios.^3,4^ Consensus recommendations for an extension of critical care service capabilities and expansion of critical care surge capacity in the face of pandemics and other disasters have been previously developed.^5^

As surges in COVID-19 patients inch toward overwhelming the health care centers, certain elective procedures are commonly restricted or canceled with the intention of minimizing pressure on limited resources and to better prepare health care facilities for the incoming COVID-19 patients. While the net impact of a cancelation of elective procedures on releasing ICU resources remains a matter of debate,^6^ this common practice can lead to a significantly reduced need for operating room and procedural staff, including nurses and anesthesiologists.

The ensuing situation can be viewed as a mismatch of resources and needs: In some areas, health care systems are faced with an increasing number of COVID-19 patients potenitally exceeding their capacity, whereas, in other areas, health care systems are faced with widespread procedural cancelations and a drop in demands. Additionally, the demand in each area can change in the matter of weeks given the the dynamic nature of the problem. This calls for an equally dynamic “rapid response” strategy to mobilize resources – including health care providers – from areas where there is waning demand and redeployment to areas where there is waxing demand.^7^

Reports on redeploying clinicians in response to higher demands and various strategies to optimize care in the face of the pandemic are emerging. Notably, many reports to date primarily describe redeploying across specialties/wards, and there are fewer reports on deploying clinicians to other geographical areas facing increased demand. In a report from a large academic medical center in New Jersey, the vast majority of physicians employed in the Department of Medicine agreed to be redeployed to provide acute hospital care or non-face-to-face supportive roles, while challenges in personal risk, medical-legal matters, clinical competency, and financial arrangements came to light.^8^ A rural health care system in upstate New York followed these 4 strategies to address the increased caseload and shortages: (1) expansion of ICU capacity; (2) redeployment and retraining of the workforce; (3) provision of COVID-19 information, testing, and follow-up for the community; and (4) coordination of the COVID-19 response across the organization.^9^

Regulatory adjustments have further paved the way to implementing these strategies. Currently, many states in the United States have implemented policies to allow and facilitate practice by health care professionals licensed in other states. Centers for Medicare and Medicaid Services (CMS) Conditions of Participation (COP) for admission and billing have adapted to this new landscape.

Similarly, clinicians from specialties with reduced caseload (eg, surgery) can be reassigned to critical care.^10,11^ To address this need, the American Society of Anesthesiologists (ASA) in collaboration with other anesthesia and critical care professional societies have developed the COVID-Activated Emergency Scaling of Anesthesiology Responsibilities in the ICU (CAESAR-ICU) workgroup.^12^ CAESAR-ICU is intended to enable general anesthesiologists to provide limited critical care services. An added benefit of such a strategy is the potential for better preparation of the health care staff as they gain more experience in treating COVID-19 patients. This is particularly important in managing a newly emerged disease with limited evidence-based management guidelines and substantive gaps in clinical knowledge.

## TeamHealth Approach

TeamHealth (Knoxville, TN, USA) is a physician-led multidisciplinary health care organization with a team of around 20 000 affiliated clinicians practicing in emergency medicine, hospital medicine, anesthesiology and critical care, specialty hospital care, ambulatory care, and post-acute care in most states across the United States. TeamHealth rolled out a systematic and organized approach to optimize the response to the moving waves of COVID-19 across states while tapping into its less utilized resources.

COVID-19 information channels were added to TeamHealth existing online, mobile-first, Health Insurance Portability and Accountability Act (HIPAA)-compliant communication app platform (Zenith®), allowing the rapid dissemination of up-to-date, evidence-based treatment information gained through national and international clinical experiences. The provision of personal protective equipment (PPE) was addressed in partnership with existing and new suppliers.

Recognizing the magnitude and indefinite duration of this calamity, clinical and administrative staff were assembled into clinical and administrative task forces to assess the impact of reduced demand and cancelation of elective procedures from multiple perspectives:

- Projection of decreased patient procedural volumes and associated revenue reductions
- Assessment of redeployment strategies for those faced with reduced clinical work to areas of increased clinical need, as well as acceptable remuneration models for various scenarios, for example, non-anesthesia-related practice performed by anesthesia staff
- Review of the current regulatory environment to assess the ability to rapidly deploy clinicians across state lines for clinical practice
- Creation of processes for dissemination of this information to guide clinical staff deployment in *real time*

## Clinical Support

An organization-wide multispecialty Emerging Infectious Diseases task force was established early on as the pandemic was declared. The task force prepared a whitepaper for TeamHealth clinicians to assist the task force with awareness and preparedness.

The task force represented physicians and advanced practice clinicians (physician assistants and nurse practitioners) in various specialties, including emergency medicine, hospital medicine, critical care, and anesthesiology. The task force evaluated and addressed COVID-19-related clinical challenges, including deployment, ethical issue of resource distribution, infectious disease management, critical care for non-critical care clinicians, human resource adjustments, and health care workers’ well-being and mental health (including burnout and stress and anxiety management).^13^ Discussions were held via remote video conference calls on a daily and weekly basis to identify and address ongoing clinical and operational issues. The task force reviewed the process and flow of care from intake to discharge, promulgated established standards of care and minimum requirements (eg, for proning and intubating teams), and assembled accumulating clinical data from various sources across the globe on the management of COVID-19.

These efforts led to the creation of a series of consensus statements/guidelines and educational modules, including guidelines for airway management teams, supporting emergency departments (EDs), and redeploying other specialties to critical care. Other consensus statements addressed PPE policies and needs, early intubation, non-invasive ventilation modes, use of emerging therapeutics, repurposing anesthesia machine ventilators for use in the critical care setting, volume resuscitation, new interventions and therapeutics, education for non-critical care staff on current critical care matters, and ethical considerations.

These resoures have been undergoing revisions and updated frequently, and disseminated among the clinical teams through various channels, including Zenith® postings (eg, clinical alerts and videos) and town hall meetings. The resources have also been made publicly available to other clinicians and health care facilities, as well as through our website (https://www.teamhealth.com/covid-19/). Examples of documents publicly available include COVID-19 and Critical Care Resource Allocation, COVID-19 Critical Care FAQs for Non-Critical Care Clinicians, COVID-19 and Pregnancy, Hospital Incident Command and Pandemics, Guidance for CODE Blue and Emergent Medical Response in COVID-19 Patients, Guidelines for Airway Management of Suspected or Confirmed COVID-19 Patients, COVID-19 Intubation Pre-entry Checklist for Providers, COVID-19 Intubation Rotation, and Termina Extubation.

## Managing the Deployment

In response to the mismatch of resources and needs across various areas, 3 main deployment scenarios were considered:

1. Within the current institution (eg, from operating rooms to dedicated intubation teams and critical care units)
2. Within the organization (from 1 TeamHealth client facility to another in any clinical service line)
3. Redeployment from a TeamHealth client facility to a non-client facility

**Figure 1.**
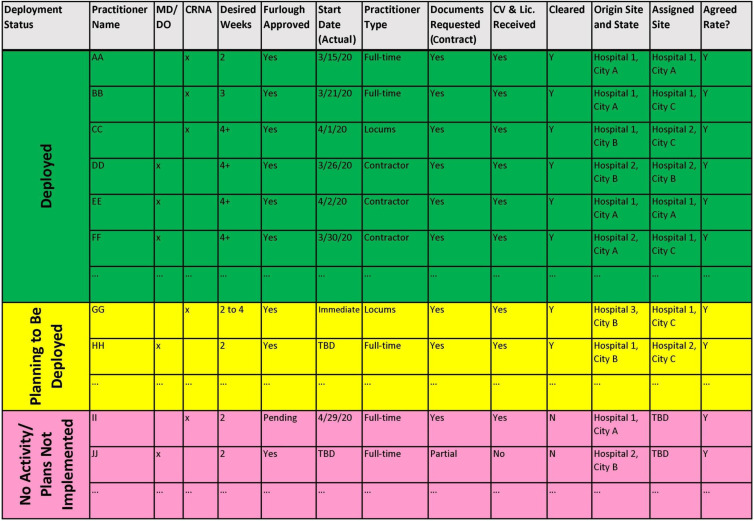
A sample of the color-coded grid used to visualize and track deployment of clinicians. CRNA = certified registered nurse anesthetist; CV = curriculum vitae; DO = Doctor of Osteopathic Medicine; MD = Doctor of Medicine.

The deployment task force established “rules of engagement” for these scenarios. A color-coded grid of anesthesia teams across TeamHealth-contracted hospitals was created to allow real-time visualization of deployments in progress, identifying each clinician’s status as “deployed,” “planning to be deployed,” and “no activity/plans not implemented.” The grid also contained information on the numbers of physicians and certified registered nurse anesthetists who were or would need to be furloughed at their home institution (Figure 1).

The hospitals in need of deployment were queried to identify the needed service line(s); type and number of practitioners needed; needed schedules and start and end dates; information on the work location and setting (intubation teams, ICU, ED); patient mix (COVID-19 and non-COVID-19); PPE availability; administrative elements, such as health record systems; and verification of licensing and credentialing requirements and insurance coverage. This information was supplied to the TeamHealth headquarters for contract negotiation and approval, and a “demand” grid was created to visualize the hospitals’ need for coverage in real time.

The information in the grids was used to provide local deployment recommendation to the idle staff (Scenario 1), to review the ability of idle staff to deploy to other TeamHealth-client hospitals (Scenario 2), and to create a pool of locum tenens clinicians to deploy to other areas without a TeamHealth-client relationship (Scenario 3). To further facilitate the deployment process, clear and concise checklists were created for the clinicians to be deployed and the requesting institutions, so that the clinicians would know what to expect and what service they were expected to provide once deployed. Educational modules were prepared and provided to the clinicians. While managing deployments, the task force continued monitoring the current and expanded ICUs in TeamHealth-contracted centers for the number of ICU beds, ventilators (from ICU and repurposed from operating rooms), and current staffing to ensure that capacity to respond to demands at various areas was not compromised.

## Conclusion

The COVID-19 pandemic has posed many health care systems across the world with unprecedented challenges. Widespread community prevalence and high susceptibility to COVID-19 and its relatively high contagiousness have the potential of creating perfect storms of volatile surges of new cases exceeding local capacity.

Drawing on its wide geographic footprint and outreach, operational expertise in human resource, information technology, legal and contracting, and financial management, TeamHealth has been able to use this challenge as an opportunity to redeploy clinicians from areas of less demand to areas outside of their normal practice facing immediate need. This was made possible through a systematic approach carried over by various task forces assembled by clinicians and administrative staff. Clinical support was provided through consensus statements and guidelines that were continuously updated to reflect the latest on management strategies for COVID-19. Deployments were managed using a grid system to identify the clinicians available and areas in need, allowing the 2 to be matched efficiently. While we were able to successfully deploy our clinicians to provide service in areas of more demand during the pandemic, and we have received positive anecdotal feedback from the deployed clinicians and the hospitals where they have been deployed, more research is needed to assess the clinical impact of this endeavor in terms of case volumes managed by the deployed clinicians.
